# From Lab to Field: Damp Heat Testing and its Implications for PV Module Service Lifetime

**DOI:** 10.1002/gch2.202400229

**Published:** 2025-03-24

**Authors:** Abdulkerim Gok

**Affiliations:** ^1^ Department of Materials Science and Engineering Gebze Technical University Gebze/Kocaeli 41400 Turkey

**Keywords:** damp heat, degradation, photovoltaic modules, service lifetime, testing time

## Abstract

Damp heat testing, as outlined in the IEC 61215 standard, evaluates PV modules under prolonged humidity exposure. However, its effectiveness as a reliability test remains debated. This study maps damp heat testing times equivalent to a service lifetime of 30 years across Europe, highlighting the impact of local climate conditions. The activation energy of power degradation is key in determining testing times. For an activation energy of 0.6 eV, testing times range from over 2250 h in southern Europe to <750 h in northern Europe. In central Europe, testing times generally range from 1250 to 1750 h. Madrid and Rome stand out among major European capitals for their testing times, with Madrid having one of the shortest and Rome one of the longest. In Madrid, increasing the activation energy from 0.4 to 0.8 eV reduces the testing time from 5548 h to just 290 h. To ensure the standard testing time of 1000 h represents a service lifetime of 30 years across Europe, the activation energy should be at least 0.7 eV. These findings emphasize the need for customized testing protocols that consider regional climate differences and design factors, rather than relying on a one‐size‐fits‐all approach.

## Graphical abstract

Growing global concerns about climate change are boosting PV system installations; however, it is important to acknowledge the durability and reliability issues PV modules encounter during real‐world operation. This study maps the equivalent damp heat testing times for a service lifetime of 30 years across Europa. It emphasizes the significant impacts of local climate conditions and module constructions on testing times, highlighting the risks of relying on standard testing for service lifetime estimation.

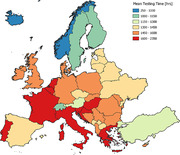

## 1 Introduction

Solar energy is pivotal in advancing the transition to a cleaner and more sustainable energy future. The global deployment of photovoltaic (PV) systems has expanded rapidly, reaching 1.6 TW of installed capacity in 2023 and projected to grow to 2.2 TW in 2024.^[^ ^1^, ^2^ ^]^ This remarkable growth is primarily driven by declining costs, the urgent need to address climate change, and the growing demand for energy security.^[^ ^3^ ^]^ Nevertheless, the economic feasibility, public acceptance, and environmental advantages of PV systems depend heavily on their field performance, affordability, and operational service lifetime. These factors are directly influenced by the durability and reliability of PV modules.^[^ ^4^ ^]^ During operation, PV modules are exposed to a wide range of environmental stressors, varying in intensity and combination, which lead to different degradation and failure mechanisms over time.^[^ ^5^, ^6^, ^7^, ^8^, ^9^, ^10^, ^11^, ^12^, ^13^, ^14^, ^15^, ^16^ ^]^ Tackling the challenges related to PV module service lifetime is crucial to supporting the sustainable and long‐term expansion of solar energy.

PV modules are composed of several critical components, each essential for ensuring their safety and performance throughout their service lifetime. Ethylene‐vinyl acetate (EVA), a widely used encapsulant, is prone to hydrolytic degradation, which generates acetic acid as a byproduct. This acid accelerates cell metallization corrosion, increases series resistance, and leads to power losses, ultimately shortening the service lifetime of modules.^[^ ^17^, ^18^, ^19^, ^20^, ^21^ ^]^ The degradation process is further accelerated by elevated temperatures. To enhance the long‐term performance of PV modules, careful selection of bill‐of‐materials and optimization of lamination processes are crucial. Recent studies have explored alternative encapsulation materials and lamination strategies to address these challenges.^[^ ^22^, ^23^, ^24^, ^25^, ^26^, ^27^, ^28^, ^29^, ^30^, ^31^, ^32^, ^33^, ^34^ ^]^ Extending the operational lifetime by improving durability and reliability of PV systems delivers significant economic and environmental benefits. It reduces the levelized cost of electricity while minimizing environmental impacts, supporting the transition toward a more sustainable future.^[^ ^35^ ^]^

In the current market, module manufacturers typically offer a 30‐year performance warranty, guaranteeing at least 80% of the initial nameplate capacity by the end of the warranty period. However, a critical issue lies in the application of these warranties without accounting for the complexities of real‐world conditions. These conditions are inherently variable and uncontrolled, involving multiple simultaneous cyclic stress factors that vary significantly by location. Moreover, the diverse constructions of modules lead to substantial differences in their degradation behavior, further complicating accurate predictions of their service lifetimes.

Standard certification testing ensures that modules meet initial safety and performance requirements at the time of manufacturing and installation. However, these tests offer limited insight into long‐term degradation mechanisms or rates and are not designed to predict service lifetimes. Typically, standard tests apply a single set of constant stress levels over a fixed time period, which do not replicate real‐world conditions. Moreover, these tests are conducted over relatively short durations, either failing to sufficiently accelerate real‐world degradation mechanisms or introducing unrealistic ones unlikely to occur during actual operation. For instance, the damp heat test, one of the key protocols in the IEC 61215 standard, aims to assess the performance of modules under high humidity and temperature conditions.^[^ ^36^ ^]^ While this test can help identify potential manufacturing defects and hydrothermal degradation mechanisms, its relevance to real‐world performance remains a topic of debate in the PV community.^[^ ^37^ ^]^ Despite this, manufacturers often market the damp heat test as an indicator of service lifetime, even though its ability to predict long‐term field performance is uncertain.^[^ ^38^, ^39^, ^40^, ^41^, ^42^ ^]^

For instance, Wohlgemuth and Kempe argues that damp heat testing exposes modules to humidity levels far exceeding those typically encountered in real‐world conditions, raising questions about its reliability as a long‐term performance indicator.^[^ ^43^ ^]^ To address these limitations, there is a critical need to bridge the gap between standard testing protocols and real‐world conditions. Koehl et al. introduced a methodology for estimating the equivalent damp heat testing times needed to represent a targeted service lifetime under actual operating conditions.^[^ ^44^, ^45^ ^]^ This approach incorporates the activation energy of power degradation observed during testing. To emphasize the impact of varying climatic conditions, they applied this methodology to specific locations across diverse climate zones.

In this study, the methodology proposed by Koehl et al.^[^ ^44^, ^45^ ^]^ was employed to estimate the damp heat testing times required to simulate a service lifetime of 30 years. Rather than restricting the analysis to specific locations, it was extended to encompass the entire continent of Europe. The results were visualized as maps, providing a comprehensive overview of how local climate conditions influence the required testing times across the region. This mapping approach not only deepens the understanding of local climatic effects but also highlights the limitations and potential risks of relying solely on standard testing to predict the long‐term performance of various PV module designs in diverse environmental settings. By generating maps for different activation energies or module constructions, the study reveals how design choices impact degradation behavior. These findings contribute to the ongoing discussion about the suitability of current test protocols.

## 2 Analysis Workflow

### 2.1 Damp Heat Testing Time Calculation

The methodology for calculating damp heat testing times representing a targeted module service lifetime is reported in Koehl et al.^[^ ^44^, ^45^ ^]^ and also in Gok^[^ ^46^ ^]^ as follows:

(i) Calculation of module temperature by the Faiman Model^[^ ^47^ ^]^ given in Equation (1):
(1) $$T_{mod}=T_{amb}+\frac{G_{POA}}{U_{0}+U_{1}\cdot ws}$$

where *T_mod_* and *T_amb_* are module and ambient air temperatures [°C], respectively, *G_POA_* is the irradiance incident on the plane of the module [W m^−2^], *ws* is the wind speed [m s^−1^], *U*_0_ is the combined heat loss factor coefficient (25 W m^−2^ °C^−1^), and *U*_1_ is the combined heat loss factor coefficient influenced by wind (6.84 W s m^−3^ °C^−1^). This model considers a glass/polymeric‐backsheet module construction with an open‐rack installation configuration.

(ii) Calculation of surface relative humidity according to the micro‐climate model using Equation (2):
(2) $$RH\;\left(T_{mod}\right)=RH\left(T_{amb}\right)\times \frac{p_{sat}\left(T_{amb}\right)}{p_{sat}\left(T_{mod}\right)}$$

where *RH*(*T_mod_*) and *RH*(*T_amb_*) represent surface relative humidity levels at module and ambient air temperatures, respectively, and similarly, *p_sat_* (*T_mod_*) and *p_sat_* (*T_amb_*) denote saturation vapor pressures at module and ambient air temperatures, respectively. It is important to note in these analyses that relative humidity values are expressed as dimensionless ratios rather than percentages.

Saturation vapor pressure is a function of temperature and can be calculated using the empirical formula developed by Tetens^[^ ^48^ ^]^ as shown in Equation (3).

(3) $$p_{sat}=w\times 10^{\left(\frac{T\times u}{T+v}\right)}\;$$

Here, *p_sat_* represents the saturation vapor pressure [mbar] and *T* denotes the temperature [°C]. The reference saturation vapor pressure at 0°C, *w*, is defined as 6.1078 mbar. The coefficients *u* and *v*, derived from model fitting, are given as 7.5 and 237.3 °C, respectively.

(iii) Calculation of effective surface relative humidity according to the sigmoidal model using Equation (4):
(4) $$RH_{eff}=\frac{1}{\left[1+98\times \exp\left(-9.4\cdot RH\left(T_{mod}\right)\right)\right]}\;$$

where the factor 9.4, extracted from the model fit, depends on various factors, such as the characteristics of the module materials, particularly the water solubility of the encapsulant, the permeability of the backsheet and front glass layers, and degradation mechanisms.^[^ ^44^, ^45^ ^]^

(iv) Determination of the frequency of effective surface relative humidity at the 85% level based on varying module temperatures by calculating time periods of Δ*t*_85_ using Equation (5):
(5) $$\Delta t_{85}\;\left(85\%RH,T_{mod}\right)=\Delta t\times \frac{RH_{eff}}{0.85}$$

where Δ*t* is the observed time periods. Here, the module temperature, influenced by the balance between the ambient air temperature, the heat generated by the module, and the heat dissipated to environment, is crucial in determining frequency distributions (Δ*t*_85_). An increase in temperature accelerates water diffusion and degradation processes, but reduces the effective surface relative humidity, thereby diminishing humidity's impact on degradation. Consider the daily cycles: in the morning, dew or high humidity wets the modules due to relatively low temperatures; however, in the afternoon, the absorbed solar energy raises the temperature of the modules, drying their surfaces. Consequently, this alters the frequency distributions of effective surface relative humidity levels, as detailed in Section 3.1.

(v) Applying time transformation to temperature of 85°C and integration according to the Arrhenius‐type relationship given in Equation (6) to determine testing times for a targeted service lifetime:
(6) $$\Delta t_{ref}=\sum_{1}^{N}\Delta t_{i}\cdot \exp\left[\left(-\frac{E_{a}}{k_{B}}\right)\left(\frac{1}{T_{i}}-\frac{1}{T_{ref}}\right)\right]\;$$

where Δ*t_ref_* is the testing time [hrs] at 85°C and 85% relative humidity, Δ*t_i_* is the time interval [hrs] observed in real‐world in one‐year duration, *E_a_* is the activation energy for power degradation [eV], *k_B_* is the Boltzmann constant (8.617  ×  10^−5^ eV K^−1^), *T_i_* is the temperature [K] at the time interval of Δ*t_i_*, and *T_ref_* is the temperature [K] at the standard testing conditions. Here, Δ*t_ref_* is assessed for one‐year duration, and therefore, can be extended by multiplying it by the intended service lifetime. This approach assumes that the variation in climate conditions from one year to another over the entire service lifetime is deemed insignificant. In Equation (6), the activation energy serves as a variable parameter, allowing for an exploration of its impact on the required testing times. It is important to note that this Arrhenius‐type relationship model is better suited for modules with a glass/backsheet construction as degradation in modules with a glass/glass construction is predominantly influenced by diffusion processes.

Koehl et al.^[^ ^44^, ^45^ ^]^ performed an in‐depth analysis using four distinct models to evaluate the impact of relative humidity on the degradation process:

1. ***Ambient Relative Humidity Model***: This model represents relative humidity under ambient conditions, reflecting the overall humidity levels in the environment surrounding the module. For this model, relative humidity data was directly extracted from the Typical Meteorological Year (TMY) dataset.
2. ***Micro‐Climate Model***: This model focuses on the relative humidity at the module surface, considering the local micro‐environment influenced by the module's temperature. Unlike ambient relative humidity, the module temperature alters the relative humidity at the surface, as described by Equation (2).
3. ***Squared Micro‐Climate Model***: This model incorporates the squared relative humidity at the module surface to account for hydrolysis kinetics in polymeric materials, as some materials degrade according to second‐order kinetics^[^ ^49^ ^]^ with respect to water content. It is derived by squaring the surface relative humidity calculated from Equation (2).
4. ***Sigmoidal Model***: This model represents the effective relative humidity at the module surface, designed to emulate the sigmoidal water sorption isotherms observed in polymeric materials. By using this model, a more accurate representation of relative humidity content within the module construction is achieved, particularly for degradation processes. The sigmoidal model, calculated using Equation (4), was found to be the most effective in capturing the impact of effective surface relative humidity on degradation.^[^ ^44^, ^45^ ^]^

This approach provides a framework for translating real‐world conditions into standard damp heat conditions. In estimating module temperature, it accounts for key stress factors such as ambient air temperature, irradiance, and wind speed. Similarly, in assessing relative humidity at the module surface, it treats humidity as an integrated factor to evaluate its overall impact. The analysis begins by examining the frequency of effective surface relative humidity at the 85% level as a function of module temperature for a given location. This is followed by calculating acceleration factors based on the activation energy of power degradation under damp heat conditions. Unlike previous studies that focus on specific locations within distinct climate zones, this study takes a broader approach by developing comprehensive continental maps of Europe. The goal is to gain a deeper understanding of how climate conditions influence the required damp heat testing times for a targeted service lifetime. Specifically, it seeks to assess whether the standard testing time of 1000 h adequately reflects a service lifetime of 30 years. Furthermore, it offers valuable insights into how heat and humidity affect modules with varying constructions, or activation energies, under diverse environmental conditions. It is important to note that standard tests typically address isolated degradation factors under controlled stress levels. However, the actual service lifetime of a module is influenced by the nature and intensity of real‐world stressors, the properties of its materials, its design, and its installation configuration.

### 2.2 Data Processing

For calculating humidity frequency profiles and damp heat testing times at specific geographical locations, TMY data was sourced from the Photovoltaic Geographical Information System (PVGIS)^[^ ^50^ ^]^ database. The TMY dataset is generated by selecting the most representative month for each calendar month over the entire available period (i.e., 19 years from 2005 to 2023) for PVGIS‐SARAH3 database. The data features a high spatial resolution of 0.1° for both longitude and latitude, corresponding to ≈11 and 8.6 km on the ground, respectively. This fine resolution, spanning latitudes from 34.50 to 71.50°N and longitudes from 25°W to 45°E, covers over 140 000 coordinate points across Europe, ensuring precise spatial representation. The TMY dataset provides comprehensive information for analyzing annual climate conditions, including hourly values for global horizontal, direct normal, and diffuse horizontal irradiance, relative humidity, ambient air temperature, wind speed and direction, atmospheric pressure, and downwelling longwave radiation. To calculate the plane of array irradiance at each geographic location, a crucial step in determining module temperatures, the optimal module tilt and azimuth angles were also retrieved from the PVGIS database for each coordinate. The “pvlib” Python package was employed for both the plane of array irradiance and module temperature calculations.^[^ ^51^ ^]^

For each geographical coordinate, the sourced data was meticulously checked to identify and address issues such as missing or corrupted entries. Once data quality was verified, the frequency distribution of 85% relative humidity was analyzed for a specific coordinate as a function of module temperature. Based on this analysis, testing times corresponding to a service lifetime of 30 years were calculated, considering various activation energy values. The resulting dataset includes three key variables: latitude, longitude, and the testing time associated with each activation energy. According to the study by Koehl et al.,^[^ ^45^ ^]^ activation energies for modules with EVA encapsulants and polymeric backsheets typically range from 0.5 to 0.7 eV. For mapping purposes, an activation energy of 0.6 eV was selected as a representative value for standard module constructions. Additionally, maps for modules with activation energies ranging from 0.3 to 0.9 eV are provided in the Supporting Information.

### 2.3 Interpolation and Visualization for Mapping

For interpolation, the Inverse Distance Weighting (IDW) method was identified as the most suitable approach for this dataset with a 0.1° spatial resolution, striking a balance between accuracy and computational efficiency. IDW assumes a linear relationship between data points, giving greater weight to points in closer proximity.^[^ ^52^ ^]^ This makes it a simple yet effective interpolation technique that generates a smooth, visually appealing color gradient on the interpolated map.

For visualization, the dataset was transformed into a map of Europa using the Geographic Information System (QGIS)^[^ ^53^ ^]^ tool, enabling a clear representation of spatial variations across different locations. The process began by importing the dataset into QGIS along with the shapefile^[^^]^ of Europe. To ensure accurate spatial representation, the appropriate coordinate reference system was applied. Interpolation was performed with a power constant of four, optimizing the influence of nearby data points on interpolated values. The pixel resolution for both the X and Y axes was set to 0.05 to achieve the desired level of precision. Using the shapefile, the interpolated layer was clipped to exclude regions outside the continent's boundaries, keeping the visualization focused solely on the target area. A single‐band pseudo‐color render type with a color ramp was applied to represent variations effectively. To enhance the map's detail, data interpolation was discretized for finer value representation, and early resampling methods were employed to improve rendering efficiency.

## 3 Results and Discussion

To illustrate the analysis process, as outlined in Section 2, twelve major capital cities situated across various regions of Europe were chosen. Geographical information and the Köppen–Geiger^[^ ^54^ ^]^ climate classification for each capital city is provided in the Supporting Information. These cities serve as prime examples reflecting a spectrum of climate conditions influenced by their geographical locations. For these twelve locations, Section 3.1 examines the humidity profiles and Section 3.2 explores the effect of activation energy on the required testing times. Subsequently, Section 3.3 delves into a detailed mapping discussion, emphasizing the effect of local climate conditions and activation energies on the testing times. Section 3.4 presents a sensitivity analysis that highlights the crucial significance of experimental study design in accurately determining activation energies of power degradation under damp heat exposure.

### 3.1 The Effect of Climate Conditions on Humidity Profiles

**Figure 1:**
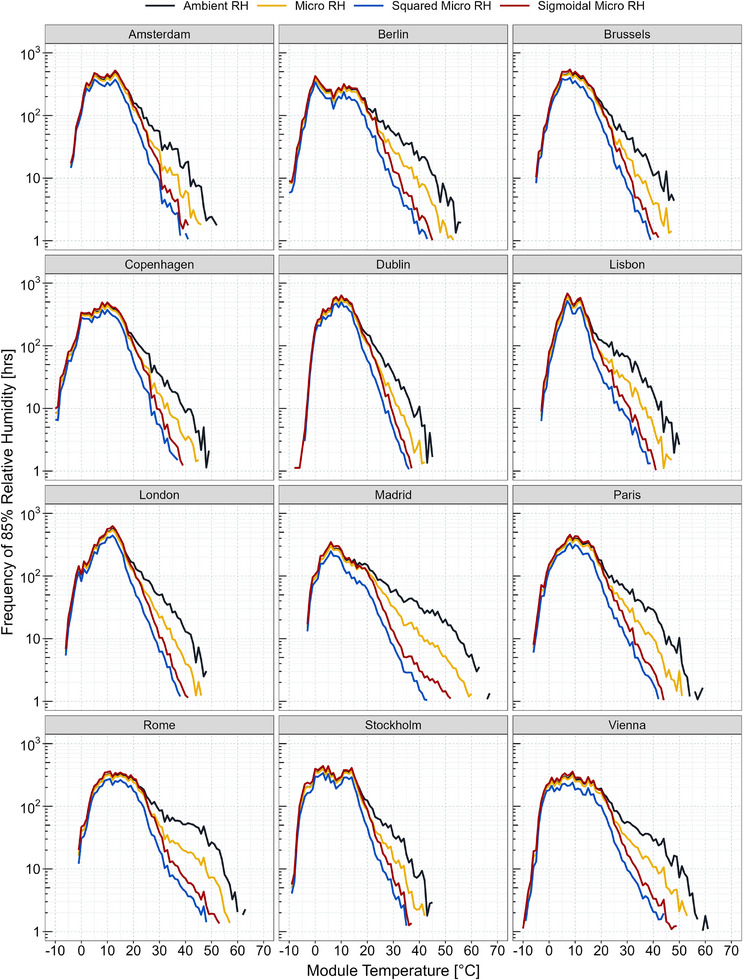
Frequency distributions of 85% relative humidity as a function of module temperature for four distinct (ambient, micro, squared‐micro, and sigmoidal‐micro) relative humidity models across chosen capital cities, showing regional variations, with distinct patterns in arid and humid regions, due to local climate conditions.

**Figure** **1** shows how frequency distribution of 85% relative humidity varies with module temperature across different humidity models applied to chosen capital cities. Analysis across diverse geographical regions highlights distinct patterns in both arid and humid locations, emphasizing the importance of local climate conditions. For instance, London exhibits notably higher frequency levels, whereas Madrid displays considerably lower frequency levels. Here, temperature is a key factor in determining the frequency profiles for a given location. As temperature rises, humidity ingress into the module structure increases, accelerating humidity‐driven degradation processes. However, higher temperatures also reduce the effective surface relative humidity, thereby diminishing the overall impact of humidity on these degradation mechanisms. At lower module temperatures, the various humidity models generally exhibit similar behavior. However, at higher module temperatures, notable differences emerge, with the sigmoidal model displaying moderate behavior compared to the others. During daylight hours, surface humidity is generally lower than ambient humidity due to the higher temperatures caused by solar radiation. Conversely, during nighttime, radiative cooling can lead to increased humidity levels. It is noteworthy that only elevated relative humidity levels effective on the module surface have a significant impact on humidity‐induced degradation. In this case, the sigmoidal model places greater emphasis on high humidity levels while still accounting for the effects of low humidity levels. At an arid location, like Madrid, characterized by substantial fluctuations in humidity throughout the day and night due to solar heating and radiation cooling, the humidity models exhibit notable variations.

### 3.2 The Effect of Climate Conditions and Activation Energies on Testing Times

**Figure 2:**
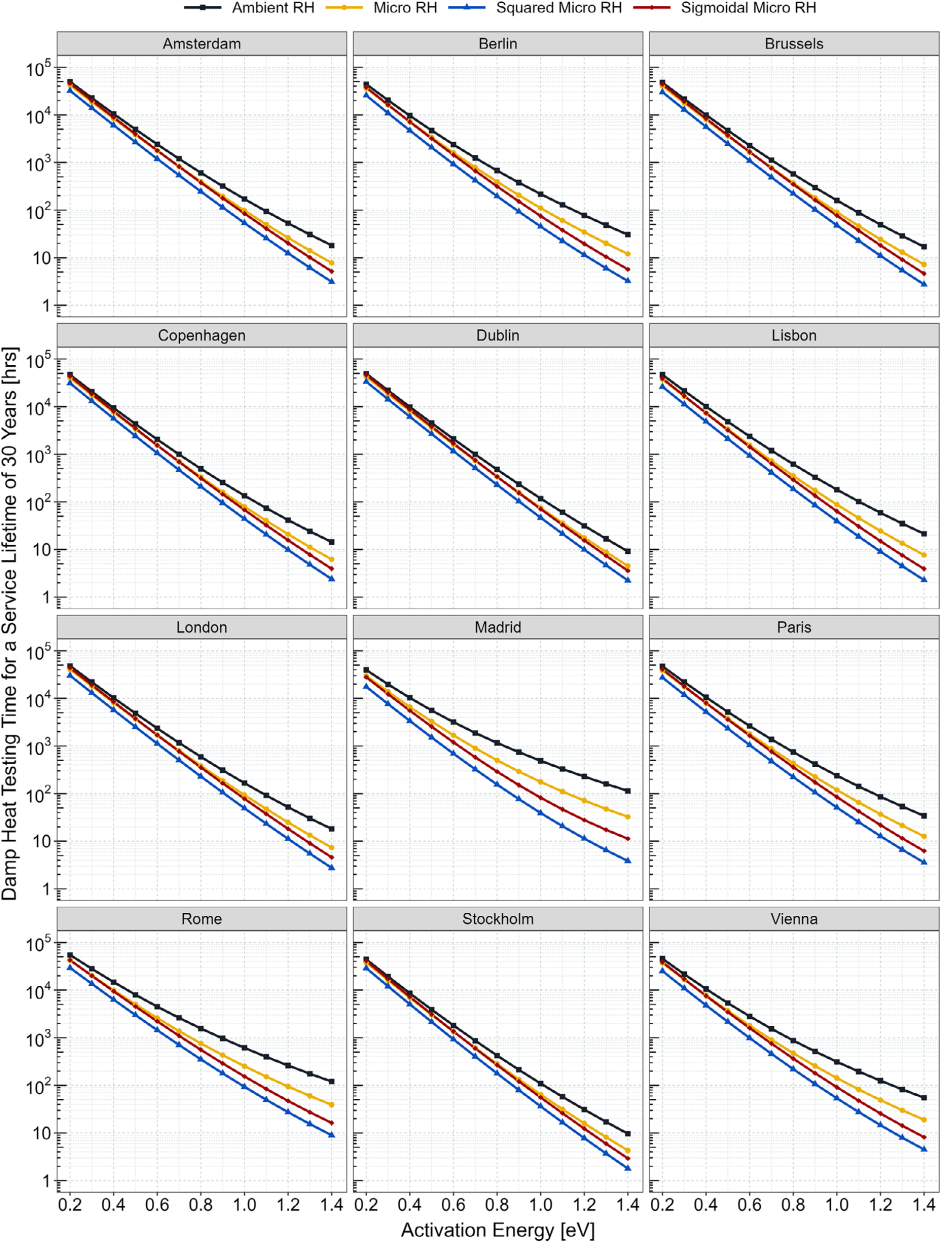
Damp heat testing times as a function of activation energy required for a service lifetime of 30 years for four distinct (ambient, micro, squared‐micro, and sigmoidal‐micro) relative humidity models across chosen capital cities, highlighting the sensitivity of testing times to activation energy, as well as the significant influence of local climate conditions.

**Figure** **2** demonstrates how testing times under damp heat exposure required for a service lifetime of 30 years varies with activation energy across different humidity models applied to chosen capital cities. Here, activation energy is treated as a free parameter. It is evident that testing times vary substantially based on activation energy due to the exponential nature of the Arrhenius relationship, indicating the importance of module construction against humidity‐driven degradation reactions. Lower activation energy leads to faster degradation kinetics, and thus, to longer testing times. Conversely, with higher activation energy, reaction kinetics slow down, resulting in shorter testing times. Another important finding is that climate conditions significantly impact testing times. Locations characterized by humid conditions tend to have longer testing times compared to drier locations. For instance, the testing time for Rome is almost twice that for Madrid for the same lifetime. Relatively higher humidity and temperature levels in Rome make it one of the locations in Europe with the longest testing time. Concerning the various relative humidity models, a similar trend to the frequency distributions is observed. In locations with humid climates, discrepancies among the models become more evident, particularly at higher activation energies, contrasting with milder, drier locations. Since the sigmoidal model was deemed the most effective at evaluating the influence of humidity on module power degradation, subsequent discussions will center around its outcomes.

**Table** **1** presents the testing times for a service lifetime of 30 years across chosen capital cities for modules with activation energies between 0.4 and 0.8 eV. In this context, the module exhibiting an activation energy of 0.4 eV denotes the most vulnerable scenario, having minimal resistance against humidity ingress. Conversely, the module characterized by an activation energy of 0.8 eV signifies the most favorable scenario, showing robust resistance against humidity ingress. Unsurprisingly, hot and humid regions require extended testing times as opposed to drier regions. For example, testing time is only 1195 h in Madrid for an average module exhibiting an activation energy of 0.6 eV for a service lifetime of 30 years. In contrast, achieving the same service lifetime in Amsterdam extends testing time to 1789 h for the same module construction. This pattern holds for other capital cities as well. Neighboring locations such as London and Brussels, both characterized by temperate oceanic climate, show very similar testing times, 1693 and 1666 h, respectively. The impact of ambient air temperature, coupled with irradiation levels and wind speed, on module temperature is apparent, thereby influencing the effective surface relative humidity levels. This emphasizes the significance of both the type and intensity of stress factors effective on the module.

**Table 1:** Damp heat testing times for a service lifetime of 30 years for modules with activation energies ranging from 0.4 to 0.8 eV across chosen capital cities.

	Activation Energy [eV]				
	0.4	0.5	0.6	0.7	0.8

Location	Damp Heat Testing Time [hrs]				
Amsterdam	8862	3959	1789	818	379
Berlin	7132	3179	1443	668	315
Brussels	8366	3712	1666	757	348
Copenhagen	7994	3492	1544	692	314
Dublin	8683	3805	1682	751	338
Lisbon	7324	3216	1427	641	292
London	8440	3761	1693	770	354
Madrid	5534	2541	1195	579	290
Paris	7985	3599	1645	763	360
Rome	9443	4536	2215	1101	559
Stockholm	7256	3116	1356	598	267
Vienna	7594	3452	1598	755	365

**Figure 3:**
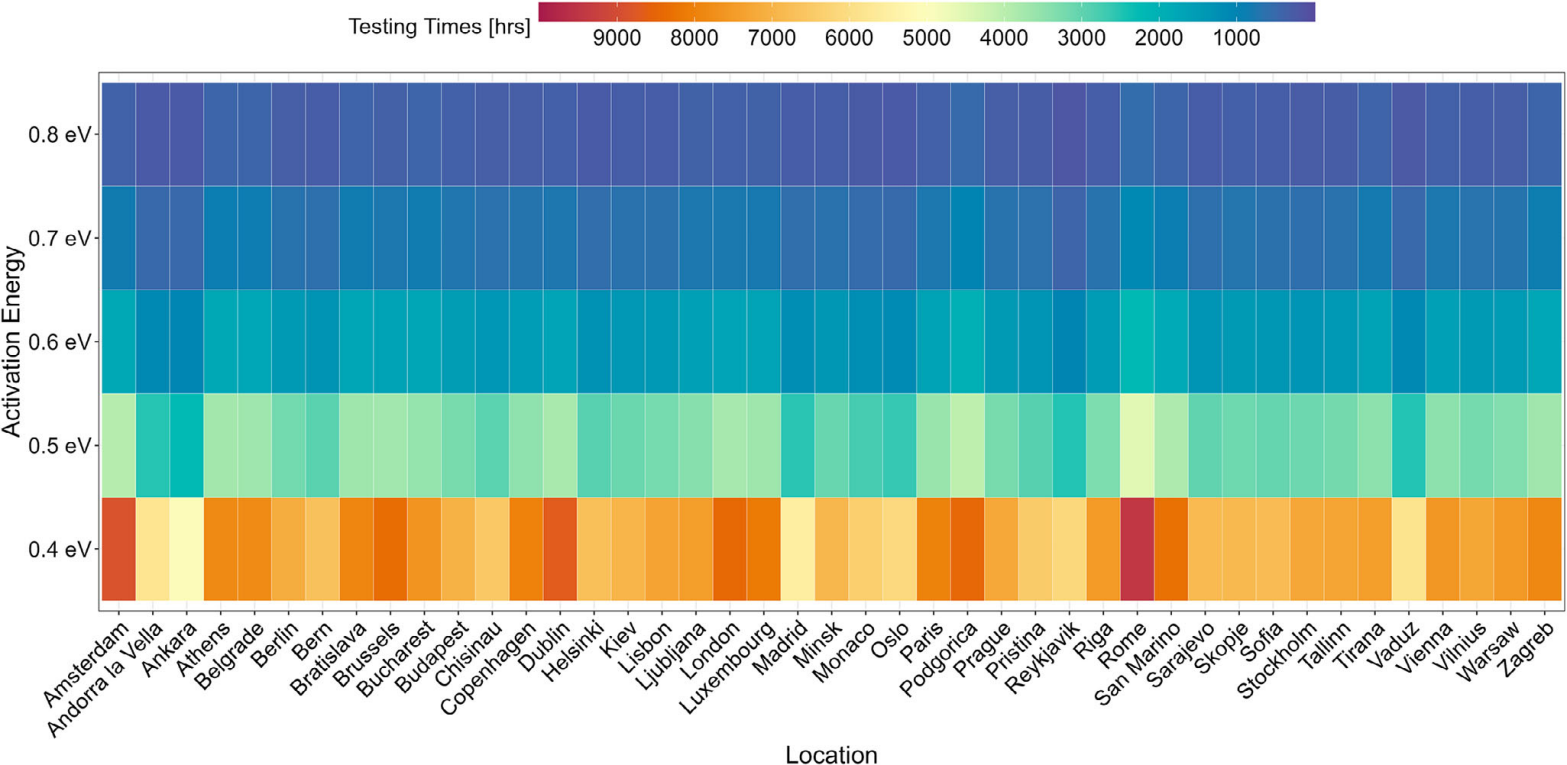
Heatmap of damp heat testing times for a service lifetime of 30 years for modules with activation energies ranging from 0.4 to 0.8 eV across all capital cities in Europe.

The testing times across all capital cities in Europe are presented visually in the heatmap in **Figure** **3** and reported in Table S2 (Supporting Information). It can be seen that the testing times vary greatly depending on the activation energy: ranging from 4000 to 10 000 h at 0.4 eV, narrowing to 600 to 2500 h at 0.6 eV, and further decreasing to 100 to 600 h at 0.8 eV. At 0.6 eV, Reykjavik, Ankara, Vaduz, Oslo, and Madrid exhibit the shortest testing times, whereas Rome, Podgorica, San Marino, Amsterdam, Athens, and Zagreb experience the longest testing times, highlighting the influence of local climatic conditions.

It is important to highlight that in case the service lifetime is intended to go beyond 30 years, the testing times listed in Table 1 (and Table S2, Supporting Information) should be modified accordingly. This modification can be accomplished via a simple transformation procedure because testing times, which are determined based on the annual acceleration factors calculated from the Arrhenius‐type relationship, exhibit a linear correlation with the desired service lifetime. For example, for a service lifetime of 40 years, testing times reported in Table 1 can be divided by 30 to determine the annual testing time, and then multiplied by 40. For instance, in Vienna, with an activation energy of 0.6 eV, the required testing time increases from 1598 to 2131 h when the service lifetime is extended from 30 to 40 years.

The results presented underline the marked impact of activation energy, essentially the resistance of module constructions to humidity ingress, on testing times. For example, Madrid, located in a country with the highest PV penetration^[^ ^1^ ^]^ with 21.1% in Europa, is characterized by its high PV power capacity, primarily attributed to favorable irradiation, dry climate conditions, and land availability. For a standard PV module with an EVA encapsulant and a polymeric backsheet (i.e., a breathable structure) having an activation energy of 0.6 eV, testing time amounts to ≈1195 h. In this situation, the standard testing time of 1000 h is close yet insufficient to represent a service lifetime of 30 years for this particular location. However, when the activation energy increases to 0.8 eV, indicating a module with a glass/glass construction equipped with an edge sealant (i.e., an unbreathable structure) for minimized humidity ingress, testing time drops significantly to ≈290 h. In this scenario, the standard testing time of 1000 h seems appropriate yet overly excessive, leading to over‐aging. In general, the activation energy for most capital cities ranges from 0.65 to 0.7 eV to be represented by the standard testing time of 1000 h. However, in locations with harsher climates, such as Rome, Podgorica, and San Marino, the activation energy should be ≈0.725 eV. Conversely, in milder climates, such as Reykjavik, Ankara, Andorra la Vella, Oslo, and Madrid, the activation energy can be ≈0.625 eV.

### 3.3 Testing Time Map of Europa

**Figure 4:**
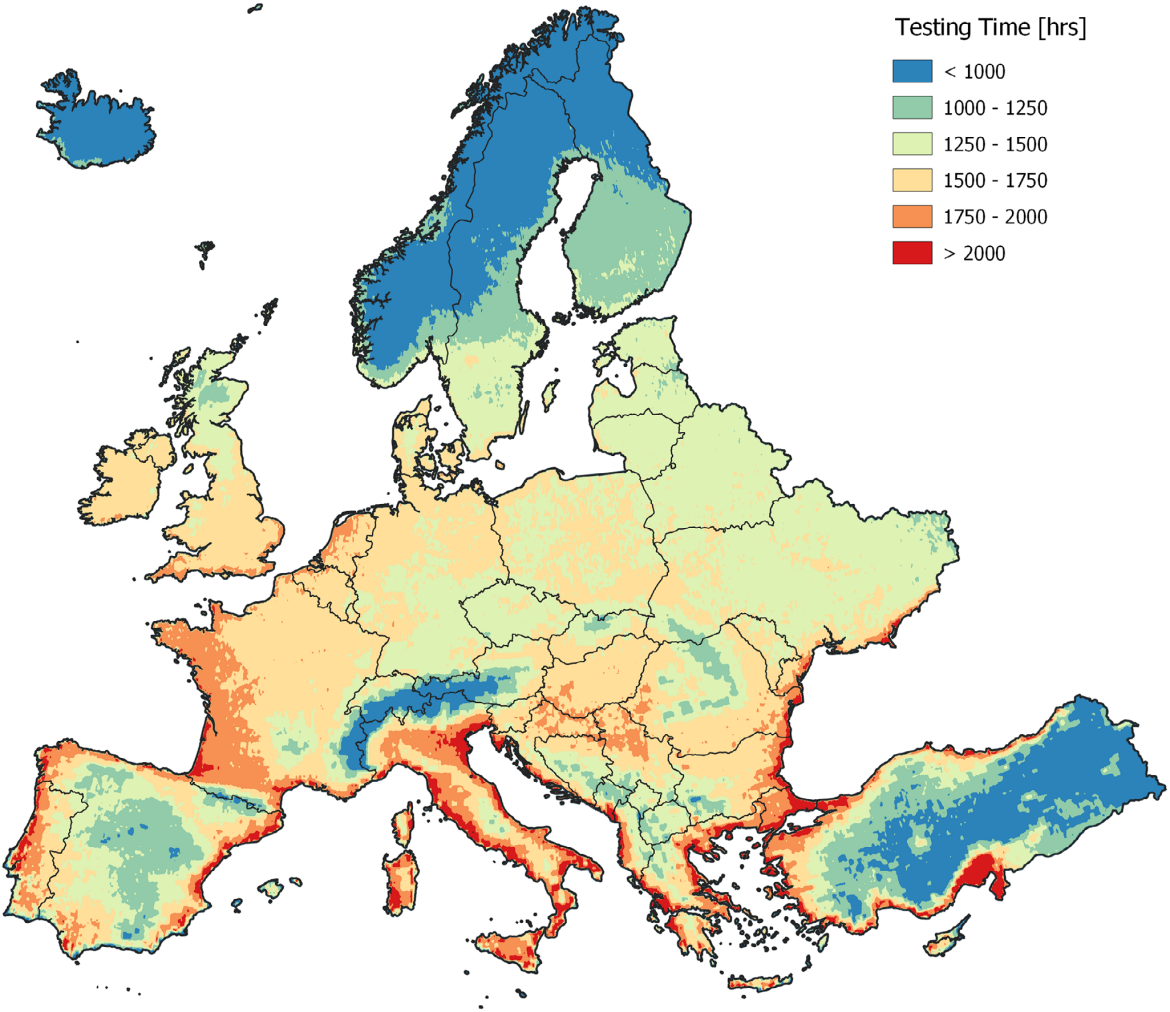
Damp heat testing time map of Europe for a service lifetime of 30 years for modules with an activation energy of 0.6 eV, showing significant variations in testing times depending on local climate conditions.

**Figure** **4** maps the testing times in Europa, representing the required hours under standard damp heat conditions. It is to be noted for the mapping process that the activation energy is assumed to be 0.6 eV for a typical glass/backsheet module to achieve a service lifetime of 30 years. The corresponding maps for modules with activation energies between 0.3 and 0.9 eV are provided in Figure S1 (Supporting Information) through Figure S6 (Supporting Information). In general, the coastal areas in the South, with higher temperature and relative humidity levels, require longer testing times compared to the coastal areas in the North and the interior regions. This is particularly true for the Mediterranean region in the South along the coasts of Turkey, Bulgaria, Greece, Albania, Montenegro, Croatia, Italy, Monaco, France, and Spain, all of which requires >2000 h of testing. The coastal areas in the North with diminished temperatures, however, require lower testing times compared to the coastal areas in the South with elevated temperatures. This is particularly true for the Baltic Sea and North Sea regions along the coasts of Finland, Sweden, Norway, Estonia, Latvia, Lithuania, Poland, Germany, Denmark, Ireland, and the Northern coasts of the United Kingdom, all of which require testing times in the range of 1250 to 1750 h. The Netherlands, Belgium, the Northern coasts of France, and the Southern coasts of the United Kingdom seem to have little higher testing times in the range of 1750 to 2000 h. Interior regions are mostly characterized with lower humidity levels than the coastal areas, requiring testing times of ≈1250 to 1750 h. However, the Alpines regions of Europe, particularly along the borders of France, Italy, Germany, and Austria, exhibit shorter testing times of <1000 h. In contrast, the area encompassing Southern Hungary, Northern Croatia, and Northern Serbia requires testing times of ≈1750 h. Meanwhile, regions to the north and south of this area, characterized with elevated terrain, have testing times of <1250 h.

In terms of country‐wise evaluation, Italy and the Netherlands are notable for having the longest testing times, while Iceland and Norway are characterized by the shortest, with average testing times of ≈1700 and 900 h, respectively. Detailed country‐specific statistics on testing times are available in Section S4 (Supporting Information). Turkey, on the other hand, with diverse geographical regions and climate conditions, appears to be the most unique country in the region, showing significant variations in testing times, ranging from 500 h in the Southeast to over 2500 h along the Southern, Western, and Northwestern coasts. A more comprehensive analysis of Turkey, covering all cities within the country, is available in the study by Gok.^[^ ^46^ ^]^

It is evident that testing times are heavily influenced by location, even within the same country, due to the substantial differences in local climate conditions. The standard testing time of 1000 h seems sufficient for capturing a service lifetime of 30 years in Iceland, parts of Norway, Finland, Sweden, Turkey, and the Alpine region for modules with an activation energy of 0.6 eV. However, it appears insufficient for nearly all other locations across Europe. This finding underlines the shortcomings of employing a single set of conditions with constant stress levels over a fixed time period, as seen in the damp heat testing, in estimating the service lifetime of modules constructed with varying packaging materials and deployed in varying climatic environments. Additionally, it emphasizes the pressing need for enhancing module constructions, particularly to withstand degradation induced by humidity.

**Figure 5:**
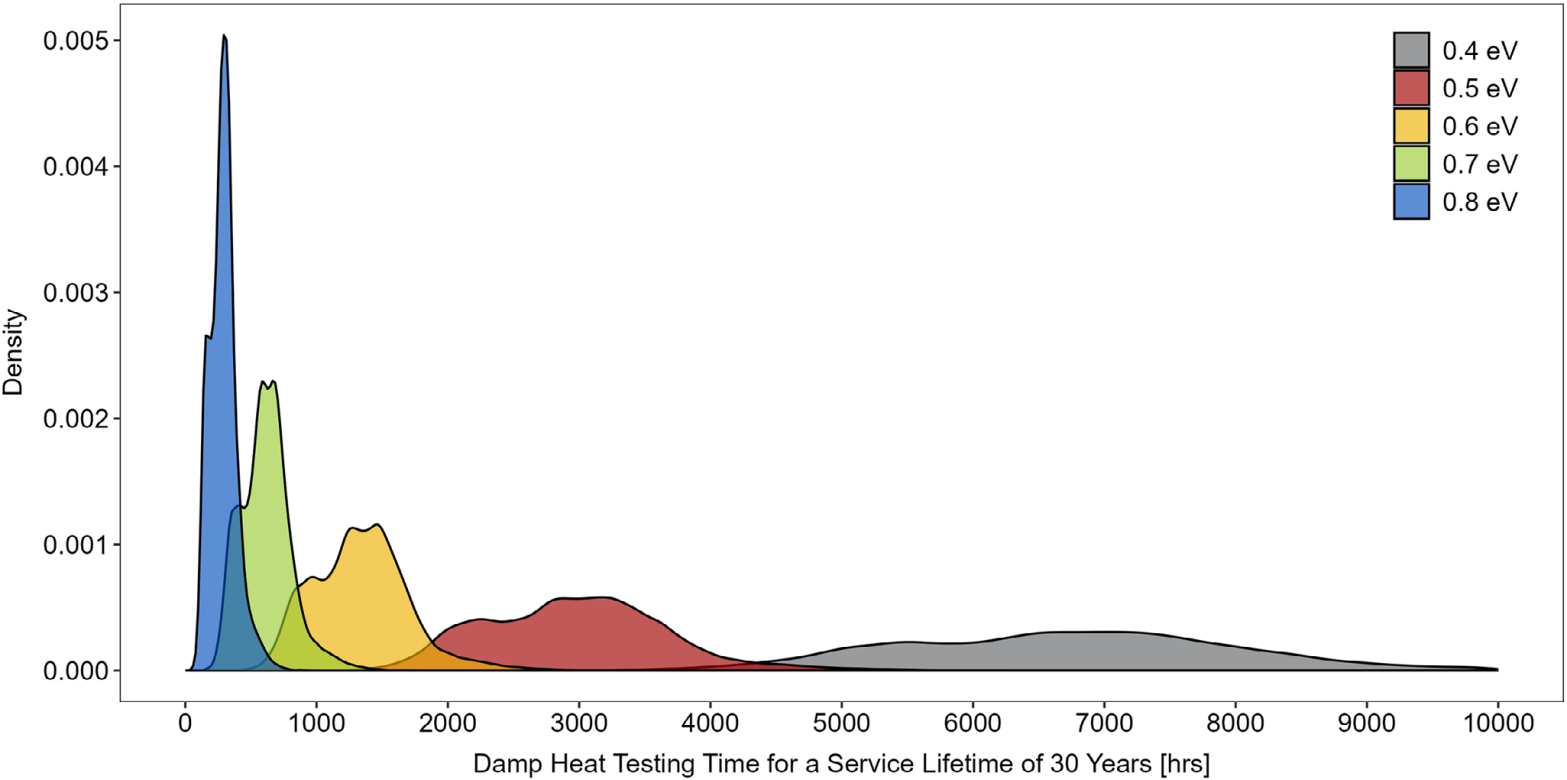
Distribution of damp heat testing times across Europe for a service lifetime of 30 years for modules with activation energies between 0.4 and 0.8 eV.

**Figure** **5** illustrates the change in distribution of testing times across Europe for activation energies ranging from 0.4 to 0.8 eV. It is evident that the population density decreases, and the distribution becomes wider, as the activation energy reduces. Irrespective of the activation energy, the overall shapes appear to be multimodal, indicating the existence of subpopulations of testing times, depending on the local climate conditions. For the distribution at 0.6 eV, the median value is ≈1330 h and modes occur at ≈966, 1273, and 1460 h, representing the Northern parts of Europa with lower, Central parts with moderate, and Southern parts with higher testing times, respectively. For this module construction, only 19.8% of the testing times fall below the standard testing time of 1000 h. When the activation energy is increased to 0.7 eV, 96.4% of the testing times are under 1000 h. Therefore, consistent compliance with the standard across Europa can only be achieved at an activation energy of above 0.7 eV.

### 3.4 Experimental Design Concerns for Activation Energy Estimations

The influence of activation energy on testing times has become apparent. Small adjustments to activation energies can lead to significant variations in testing times, owing to the exponential correlation between testing time and activation energy. For instance, reducing activation energy from 0.7 to 0.6 eV (a 14.3% decrease) results in a shift in the testing time for Amsterdam from 818 to 1789 h (a 118.7% increase). Therefore, achieving precise activation energy determination is crucial, although it presents a formidable challenge.

One approach to obtain activation energies involves conducting damp heat exposures with at least three temperature levels, such as 75, 85, and 90°C, as implemented in the study by Koehl et al.^[^ ^45^ ^]^ The Arrhenius relationship, expressed in **Equation** **7**, then allows for the calculation of acceleration factor. Time‐to‐failure (*t_f_*) is typically considered as the time when the power loss reaches 20% as commercial warranties offer 80% of the initial power at the end of warranty period. Plotting acceleration factors against the inverse temperature produces a linear relationship, from which the activation energy of the damp heat driven degradation processes can be derived by examining the slope of the constructed line. However, determining time‐to‐failure values from experimental data also involves the use of curve fitting techniques.

(7) $$\ln a=\ln\frac{{t_{f}}_{1}}{{t_{f}}_{2}}=\frac{E_{a}}{k_{B}}\left(\frac{1}{T_{2}}-\frac{1}{T_{1}}\right)\;$$

Here, *a* is the acceleration factor (or the ratio of time‐to‐failure values of *t_f_*_1_and *t_f_*_2_) when the temperature is changed from *T*_1_ to *T*_2_. To enhance reliability, conducting tests at multiple temperature conditions ensures that the degradation process remains consistent across temperature variations. The Arrhenius plot, yielding a straight line, confirms this stability, thereby enhancing the reliability of extrapolation to operational conditions for predicting service lifetime. However, this approach can be time‐ and resource‐intensive, particularly when exposures extend over long durations, such as 7000 h at 75°C.

Recent studies apply non‐linear exponential curve fitting techniques to extract activation energies without the need for multiple exposure conditions. For example, Kaaya et al.^[^ ^55^ ^]^ utilizes a power output model for hydrolytic degradation during damp heat testing as expressed in Equation (8). A review by Kaaya et al.^[^ ^56^ ^]^ also provides a valuable reference on module degradation rates and lifetime predictions as it systematically investigates different modeling approaches and assesses uncertainties and variations between them.

(8) $$\frac{P_{MPP}\left(t\right)}{P_{MPP}\left(0\right)}=1-\exp\left(-{\left(\frac{B}{\left(A\cdot RH_{eff}^{n}\cdot \exp\left(-\frac{E_{a}}{k_{B}T}\right)\right)t}\right)}^{\mu }\right)$$

Here, *P_MPP_* (*t*) and *P_MPP_* (0) are the normalized maximum power point at time t and 0, respectively, *E_a_* is the activation energy [eV], *RH_eff_* is the effective surface relative humidity level, which can be calculated using Equation (4), *n* is the indicator of the impact of *RH_eff_* on power, µ is the shape parameter of the degradation curve, *B* is the parameter related to material property, that can be set to 1 if investigating the same module construction, *k_B_* is the Boltzmann constant, *T* is the test temperature [K], *A* is the pre‐exponential constant [hrs^−1^], and *t* is the exposure time [hrs]. This equation involves five unknowns, and when extracting these using curve fitting techniques, it may lead to over‐parameterization issues, resulting in large standard errors on the parameter estimates. For example, the ratio of *B* to *A* in Equation (8) can be the same for different *B* and *A* values. To address this issue, one can perform damp heat tests at a minimum of two different temperatures while keeping the relative humidity level constant. The model then is trained using data from one temperature and tested using data from the other. However, this approach can also be time‐ and resource‐intensive. If conducting exposures at multiple temperatures is an issue, then one can iteratively predict the unknown parameters to reduce standard errors. However, without validating the results at a different temperature, the analysis would lack confidence. Once the parameters are extracted, Equation (9) can then be used to calculate the time‐to‐failure (*t_f_*) for 20% power loss.

(9) $$t_{f}=\frac{B}{A\cdot RH_{eff}^{n}\cdot \exp\left(-\frac{E_{a}}{k_{B}T}\right)\cdot {\left(\left|\log0.2\right|\right)}^{\frac{1}{\mu }}}\;$$

Regardless of the chosen approach, whether it is Arrhenius relationship or the power output model, the critical consideration lies in determining when to conclude the exposure. Both methods utilize curve fitting techniques to determine activation energies and/or time‐to‐failure values, and the chosen cut‐off time for the exposure significantly influences the final output.

**Figure 6:**
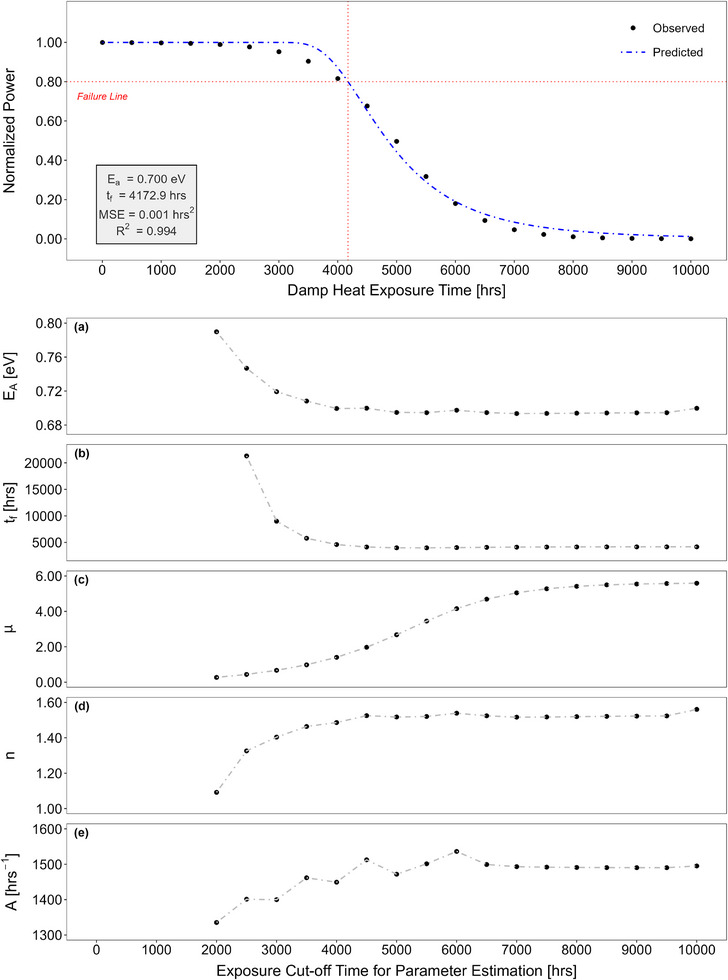
Power degradation of a hypothetical module as a function of damp heat exposure time and changes in estimated model parameters depending on the exposure cut‐off time such as activation energy (a), time‐to‐failure (b), shape parameter (c), the impact of relative humidity on power (d), and pre‐exponential constant (e).

**Figure** **6** shows the power degradation of a hypothetical module as a function of exposure time. It is a reverse s‐shaped (sigmoidal) curve with a damage accumulation period up to ≈2000 h, a subsequent reduction in power period as degradation progresses, and a saturation period after ≈8000 h. Black dots represent the experimental data, collected at intervals of 500 h, the blue curve corresponds to the model prediction, and the red line shows the failure line at 80% of initial power. The power output model, given in Equation (8), yields an activation energy of ≈0.7 eV and a time‐to‐failure value of ≈4173 h when the entire 10 000 h (≈1.14 years) of exposure is evaluated. Statistical metrics of the prediction, such as the R^2^ value of 0.994 and mean square error (MSE) value of 0.001 hrs^2^, indicate almost a perfect model fit.

The subfigures in Figure 6 demonstrate how the exposure cut‐off time affects the extracted model parameters. It is to be noted that the model prediction is applied after the damage accumulation period, i.e., 2000 h. To attain an activation energy of 0.7 eV, a minimum of 4000 h of exposure appears to be necessary (Figure 6a). Between 4000 and 7000 h, minor fluctuations are observed, but after 7000 h, it stabilizes ≈0.695 eV, and finally reaches 0.7 eV at the end of 10 000 h. A parallel trend is evident in the time‐to‐failure estimation (Figure 6b). Although fluctuations are imperceptible due to scaling, a minimum of 5000 h of exposure seems to be required to stabilize estimated time‐to‐failure values. When the exposure cut‐off time is extended from 4000 to 5000 h, the change in the estimated activation energy is roughly 0.66%. Although this minor change seems to be negligible, it leads to a substantial 13.4% change in the estimated time‐to‐failure value. Depending on the shape of the degradation curve at each cut‐off time, the shape parameter (µ) varies from 0.2 to 5.6 (Figure 6c). The impact of RH on power (*n*) (Figure 6d) and the pre‐exponential constant (*A*) (Figure 6e) stabilizes after 7000 h of exposure, settling at values of ≈1.52 and 1490 h^−1^, respectively. R^2^ values, serving as a measure of goodness of fit, range from 0.965 to 0.995, indicating nearly perfect model fits at each cut‐off time. A comprehensive analysis, including statistical metrics like MSE values, is presented in Table S4 (Supporting Information).

When conducting exposures, the best practice involves conducting exposures that last until power loss reaches a saturation point. Considering time and cost constraints, exposures should definitely extend beyond the damage accumulation period and surpass the 20% power loss threshold and preferably reach the midpoint of the sigmoid. This approach ensures reliable predictions of activation energy, time‐to‐failure, and other model parameters.

## 4 Conclusion and Future Work

In this study, the damp heat testing times required to simulate a service lifetime of 30 years were determined and mapped across Europe. Significant variations in testing times were observed, highlighting the influence of local climatic conditions and module constructions. For a module with an activation energy of 0.6 eV, testing times ranged from over 2000 h in southern Europe to <750 h in northern Europe. Central regions typically required testing times between 1250 and 1750 h. The standard testing time of 1000 h was found to be insufficient to represent a service lifetime of 30 years for this module construction in most locations in Europe. Madrid and Rome are among capitals with notably distinct testing times, representing the shortest and longest times in Europe. The impact of activation energy on the required testing times was substantial, emphasizing the critical role of module construction in enhancing resistance to humidity ingress and mitigating humidity‐induced degradation. For instance, in Madrid, increasing the activation energy from 0.4 to 0.8 eV reduced the required testing time from 5548 to 290 h. Sensitivity analyses revealed that the applied exposure time in experimental studies substantially affects model predictions, leading to considerable variations in activation energy and time‐to‐failure estimations. To improve accuracy, exposure times must extend beyond the 20% power loss threshold. These findings emphasize the limitations in standard test protocols, which apply constant stress factors and levels over fixed durations, in predicting the long‐term reliability of PV modules with varying constructions.

Enhancing the performance of PV systems demands a deeper understanding of the complex interactions between module constructions, environmental conditions, service lifetime considerations, and techno‐economic factors. Future research will adopt a broader perspective, aiming to develop comprehensive, multi‐layered maps by integrating climate conformity data with additional variables, such as irradiance, module operating temperature, terrain characteristics, land usage, and proximity to grid infrastructure. This approach will optimize site selection for large utility‐scale PV installations, identifying ideal locations that maximize performance, extend operational lifetimes, and increase profitability. Ultimately, these efforts will foster the sustainable and long‐term growth of solar energy.

## Conflict of Interest

The authors declare no conflict of interest.

## Supporting information



Supporting Information

## Data Availability

The data that support the findings of this study are available from the corresponding author upon reasonable request.
