# Supplementary material for: From Lab to Field: Damp Heat Testing and its Implications for PV Module Service Lifetime
Source: Glob Chall. 2025 Mar 24;9(4):2400229. doi: 10.1002/gch2.202400229 (PMC12003212; doi:10.1002/gch2.202400229)
Supplement: Supplementary file 1 — Supporting Information [file GCH2-9-2400229-s001.pdf]

Global
Challenges
Open Access

Supporting Information

for *Global Challenges.*, DOI 10.1002/gch2.202400229

From Lab to Field: Damp Heat Testing and its Implications for PV Module Service Lifetime

*Abdulkerim Gok**

Supporting Information

From Lab to Field: Damp Heat Testing and Its Implications for PV Module Service Lifetime

A. Gok

Department of Materials Science and Engineering, Gebze Technical University, 41400, Gebze/Kocaeli, Turkey

*e-mail: agok@gtu.edu.tr

This Supporting Information is organized as follows:

Section S1 reports the geographical data and climate zones for each European capital classified according to the Köppen – Geiger system.

Section S2 provides the equivalent damp heat testing times for a service lifetime of 30 years for each European capital, considering PV modules with activation energies ranging from 0.3 eV to 0.9 eV.

Section S3 presents maps illustrating the equivalent damp heat testing times for a service lifetime 30 years across Europe, covering PV modules with activation energies between 0.3 eV and 0.9 eV.

Section S4 offers country-specific statistics on damp heat testing times for a service lifetime of 30 years for a module with an activation energy of 0.6 eV, emphasizing the considerable variation in testing times, even within individual countries.

Section S5 provides the results of a sensitivity analysis on activation energy and time-to-failure estimations during damp heat testing, emphasizing how the choice of exposure cut-off time influences parameter estimates.

S1. Geographical Data and Climate Zones for Capital Cities in Europe

Table S1 presents the geographical data and climate zones (according to the Köppen – Geiger climate classification system) for each capital city in the European region. Some capital cities on islands were excluded from the analysis due to concerns about data accuracy.

Climate zones were determined using the *kgc* package from the Comprehensive R Archive Network (CRAN). This package identifies climate zones based on provided latitude and longitude coordinates. For locations near climate zone boundary, the package also evaluates potential climate zones in nearby locations.

The first letter in the classification indicates the major climate (A: equatorial, B: arid, C: warm temperate, D: snow, E: polar), the second letter specifies the type of seasonal precipitation (W: desert, S: steppe, f: fully humid, s: summer dry, w: winter dry, m: monsoonal), and the third letter denotes the temperature level (h: hot arid, k: cold arid, a: hot summer, b: warm summer, c: cool summer, d: extremely continental, F: polar frost, T: polar tundra).

Table S1. Geographical information and climate zones for capital cities in Europe.

Location	Country	Latitude	Longitude	Main Climate Zone	Alternative Climate Zone(s)
Amsterdam	The Netherlands	52.35	4.92	Cfb	
Andorra la Vella	Andorra	42.50	1.52	Cfb	
Ankara	Turkey	39.93	32.87	Csb	Csa
Athens	Greece	37.98	23.73	Csa	
Belgrade	Serbia	44.83	20.50	Cfa	Cfb
Berlin	Germany	52.52	13.40	Cfb	
Bern	Switzerland	46.92	7.47	Cfb	ET
Bratislava	Slovakia	48.15	17.12	Cfb	
Brussels	Belgium	50.83	4.33	Cfb	
Bucharest	Romania	44.43	26.10	Cfa	Cfb
Budapest	Hungary	47.50	19.08	Cfb	Dfb
Chisinau	Moldova	47.00	28.85	Dfb	Cfb
Copenhagen	Denmark	55.67	12.58	Cfb	
Dublin	Ireland	53.32	-6.23	Cfb	
Helsinki	Finland	60.17	24.93	Dfb	

Kyiv	Ukraine	50.43	30.52	Dfb	
Lisbon	Portugal	38.72	-9.13	Csa	Csb
Ljubljana	Slovenia	46.05	14.52	Cfb	Dfb
London	United Kingdom	51.50	-0.08	Cfb	
Luxembourg	Luxembourg	49.60	6.12	Cfb	
Madrid	Spain	40.40	-3.68	Csa	BSk/Csb
Minsk	Belarus	53.90	27.57	Dfb	
Monaco	Monaco	43.73	7.42	Csb	Cfb/Csa
Oslo	Norway	59.92	10.75	Dfb	Cfb/Dfc
Paris	France	48.87	2.33	Cfb	
Podgorica	Montenegro	42.43	19.27	Cfa	Cfb/Csa/Dfb
Prague	Czech Republic	50.08	14.47	Cfb	
Pristina	Kosovo	42.67	21.17	Cfb	
Reykjavik	Iceland	64.15	-21.95	Cfc	ET
Riga	Latvia	56.95	24.10	Dfb	
Rome	Italy	41.90	12.48	Csa	Cfa
San Marino	San Marino	43.93	12.42	Cfb	Cfa
Sarajevo	Bosnia and Herzegovina	43.87	18.42	Cfb	Dfb
Skopje	North Macedonia	42.00	21.43	Cfb	
Sofia	Bulgaria	42.68	23.32	Cfb	Dfb
Stockholm	Sweden	59.33	18.05	Dfb	Cfb
Tallinn	Estonia	59.43	24.72	Dfb	
Tirana	Albania	41.32	19.82	Csa	Csb
Vaduz	Liechtenstein	47.13	9.52	Cfb	Dfc/ET
Vienna	Austria	48.20	16.37	Cfb	Dfb
Vilnius	Lithuania	54.68	25.32	Dfb	
Warsaw	Poland	52.25	21.00	Cfb	Dfb
Zagreb	Croatia	45.80	16.00	Cfb	

S2. Equivalent Damp Heat Testing Times for Capital Cities in Europe

Table S2 reports the equivalent damp heat testing times for each European capital, considering PV modules with activation energies for power degradation under damp heat conditions between 0.3 eV and 0.9 eV. These calculations are based on a service lifetime of 30 years using the sigmoidal relative humidity model.

Table S2. Equivalent damp heat testing times for capital cities in Europe for a service lifetime of 30 years for modules with activation energies ranging from 0.3 eV to 0.9 eV.

	Activation Energy [eV]						
	0.3	0.4	0.5	0.6	0.7	0.8	0.9
**Location**	**Damp Heat Testing Times [hrs]**						
Amsterdam	20049	8862	3959	1789	818	379	178
Andorra la Vella	13595	5789	2512	1114	506	236	114
Ankara	11579	5056	2257	1033	486	236	119
Athens	16804	7850	3717	1787	873	434	220
Belgrade	17020	7807	3663	1760	867	438	228
Berlin	16281	7132	3179	1443	668	315	152
Bern	15549	6680	2933	1319	609	289	142
Bratislava	17466	7925	3669	1734	837	414	209
Brussels	19052	8366	3712	1666	757	348	163
Bucharest	16738	7639	3562	1699	830	416	215
Budapest	15668	7025	3220	1511	727	359	182
Chisinau	14769	6506	2937	1360	647	316	159
Copenhagen	18517	7994	3492	1544	692	314	145
Dublin	19977	8683	3805	1682	751	338	154
Helsinki	15796	6718	2907	1281	574	262	122
Kyiv	15953	6951	3091	1403	651	309	150
Lisbon	16833	7324	3216	1427	641	292	135
Ljubljana	16672	7446	3394	1581	754	369	185
London	19122	8440	3761	1693	770	354	165
Luxembourg	18289	8129	3669	1683	786	374	182
Madrid	12303	5534	2541	1195	579	290	151
Minsk	15939	6913	3071	1397	650	309	150
Monaco	14907	6374	2763	1216	544	247	115

Oslo	14649	6125	2619	1145	512	234	109
Paris	17944	7985	3599	1645	763	360	173
Podgorica	17994	8459	4060	1993	1001	517	274
Prague	16559	7246	3225	1462	676	319	155
Pristina	14655	6452	2898	1332	627	304	153
Reykjavik	15321	6157	2502	1029	428	181	77
Riga	17437	7526	3303	1474	669	308	145
Rome	19950	9443	4536	2215	1101	559	290
San Marino	18143	8294	3855	1825	881	435	220
Sarajevo	15936	6851	3001	1341	612	286	138
Skopje	15382	6872	3130	1457	696	342	174
Sofia	15538	6804	3036	1383	644	308	151
Stockholm	17110	7256	3116	1356	598	267	121
Tallinn	17079	7314	3195	1425	649	302	144
Tirana	16365	7441	3455	1642	801	402	209
Vaduz	14090	5873	2503	1093	491	227	109
Vienna	16998	7594	3452	1598	755	365	180
Vilnius	16866	7272	3205	1443	664	312	150
Warsaw	17332	7548	3341	1504	689	322	153
Zagreb	17239	7888	3691	1769	870	440	230

S3. Equivalent Damp Heat Testing Time Maps of Europe

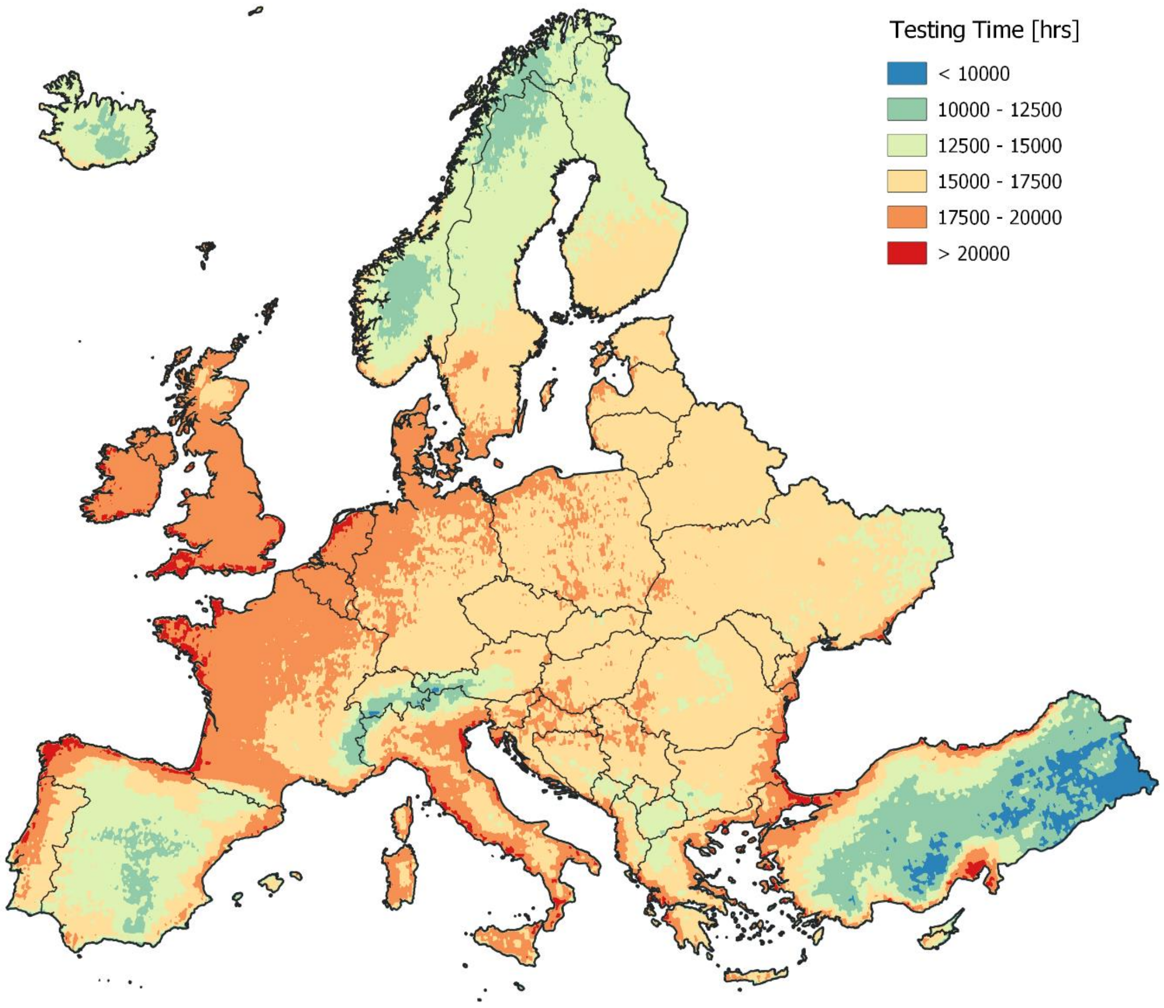

Figure S1. Equivalent damp heat testing time map of Europe for a service lifetime of 30 years for a module with an activation energy of 0.3 eV.

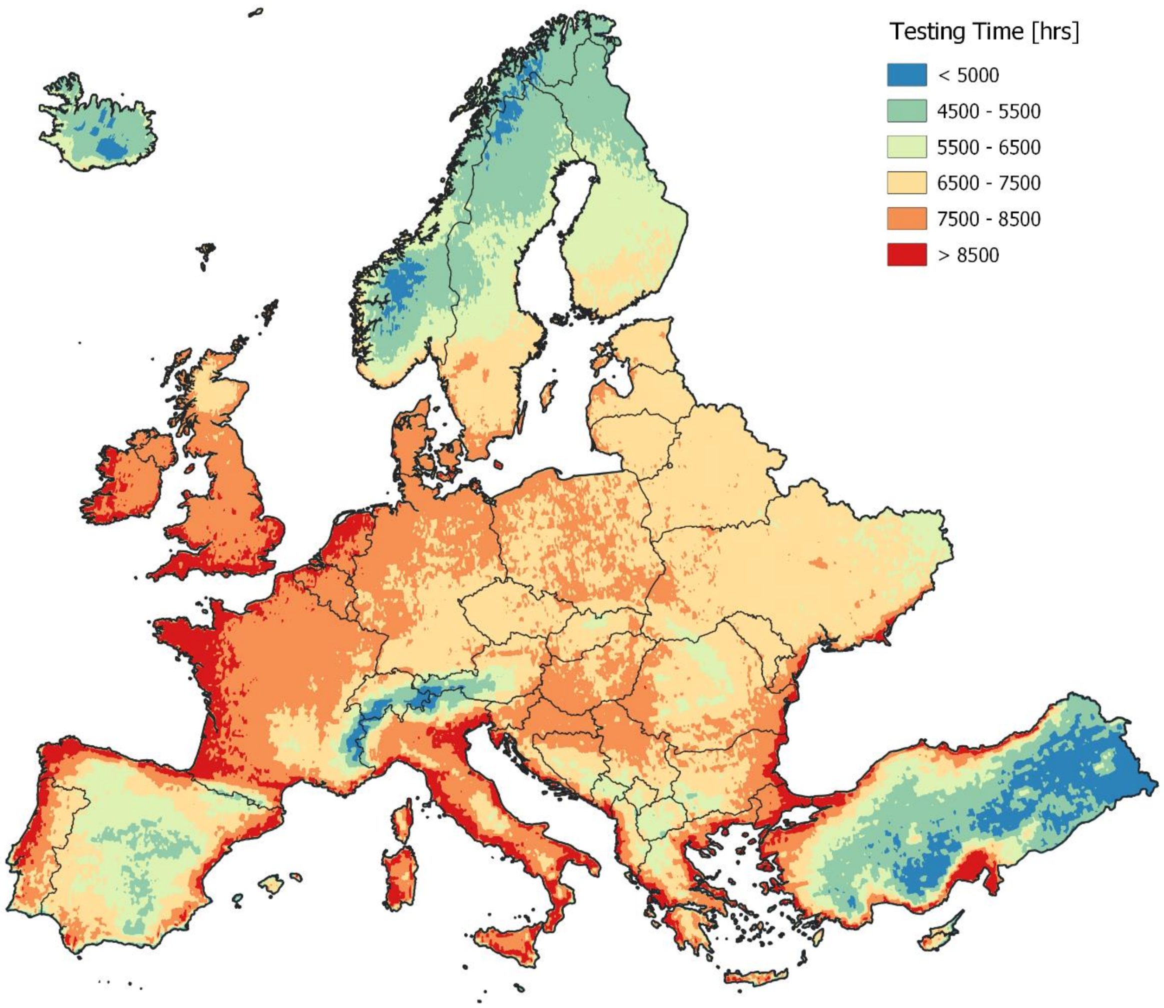

Figure S2. Equivalent damp heat testing time map of Europe for a service lifetime of 30 years for a module with an activation energy of 0.4 eV.

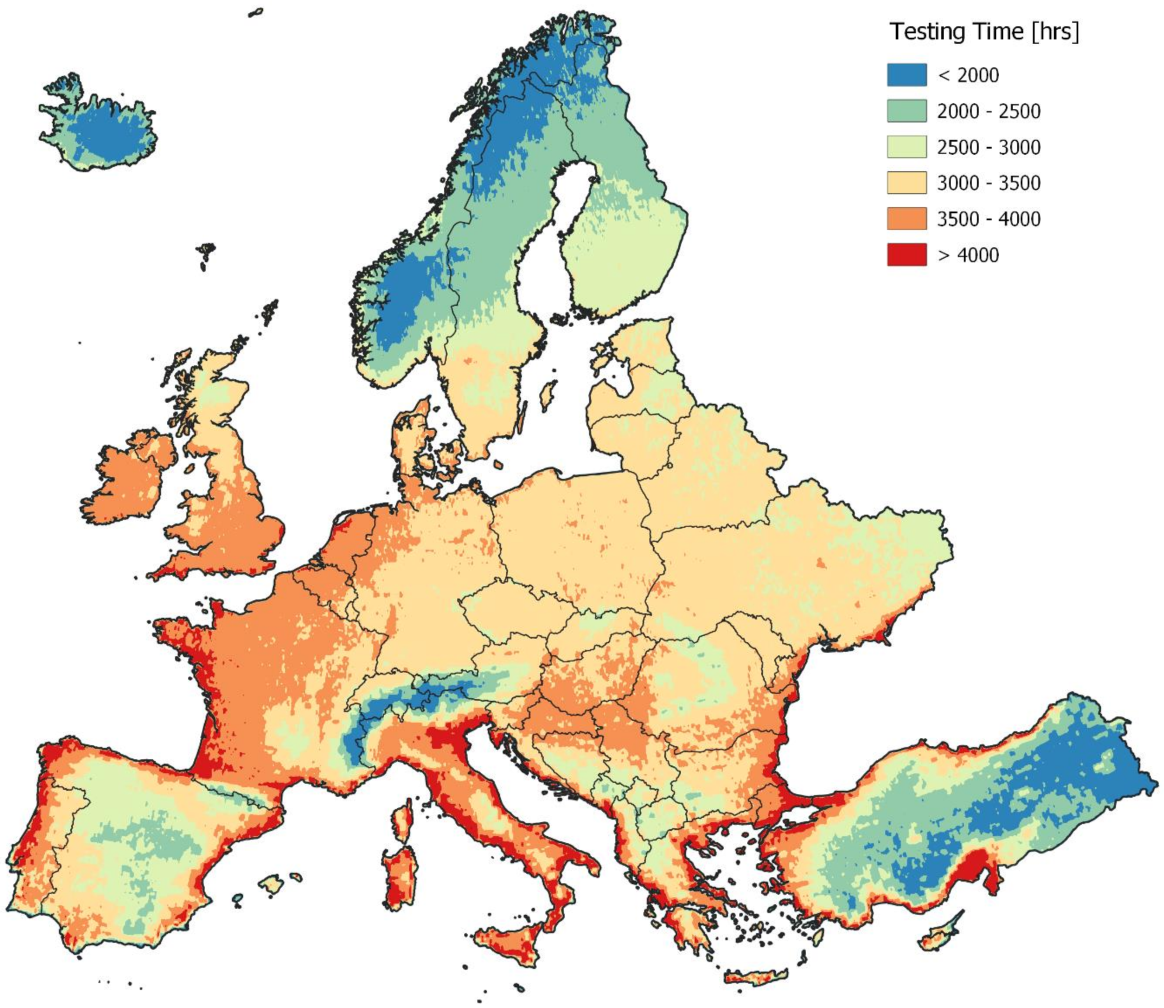

Figure S3. Equivalent damp heat testing time map of Europe for a service lifetime of 30 years for a module with an activation energy of 0.5 eV.

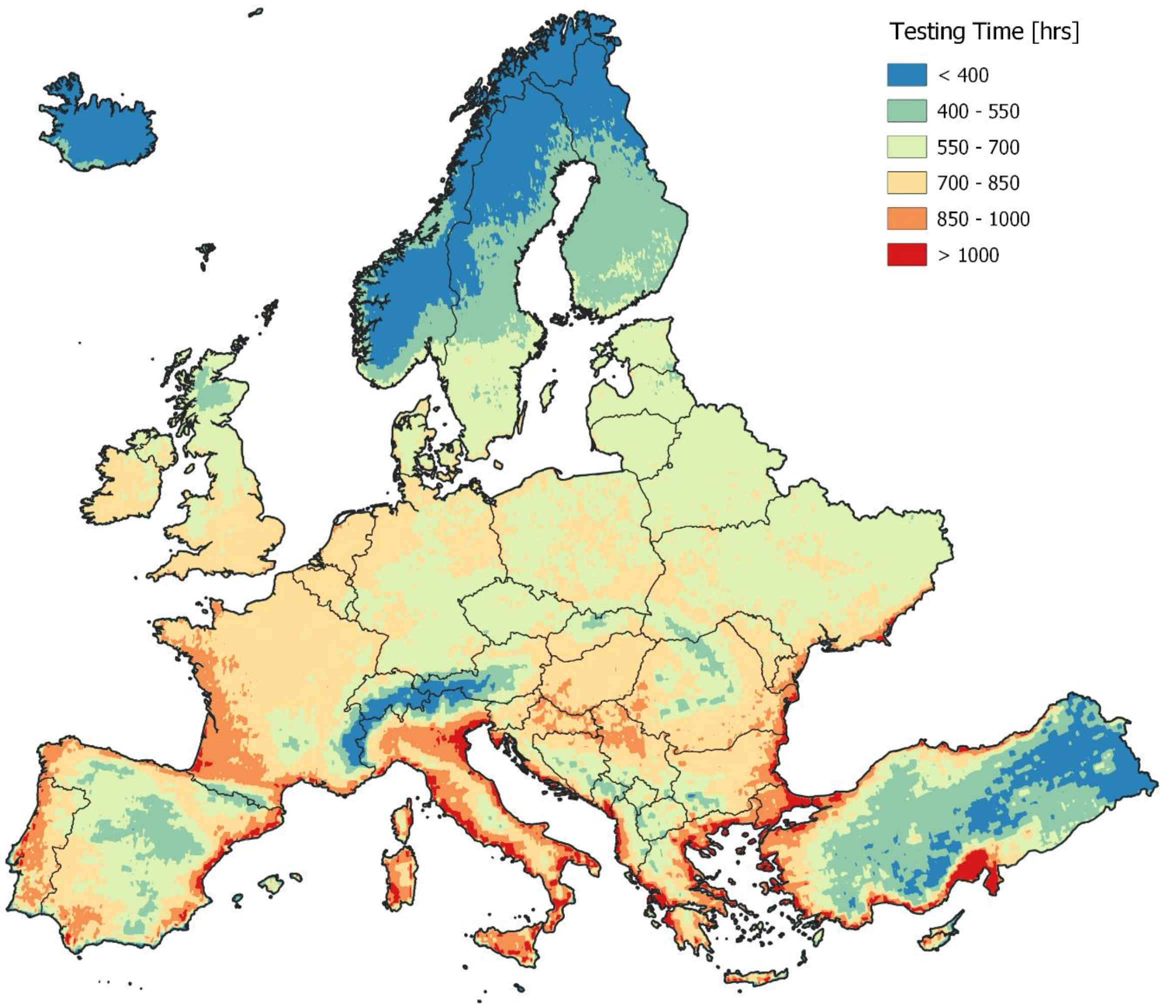

Figure S4. Equivalent damp heat testing time map of Europe for a service lifetime of 30 years for a module with an activation energy of 0.7 eV.

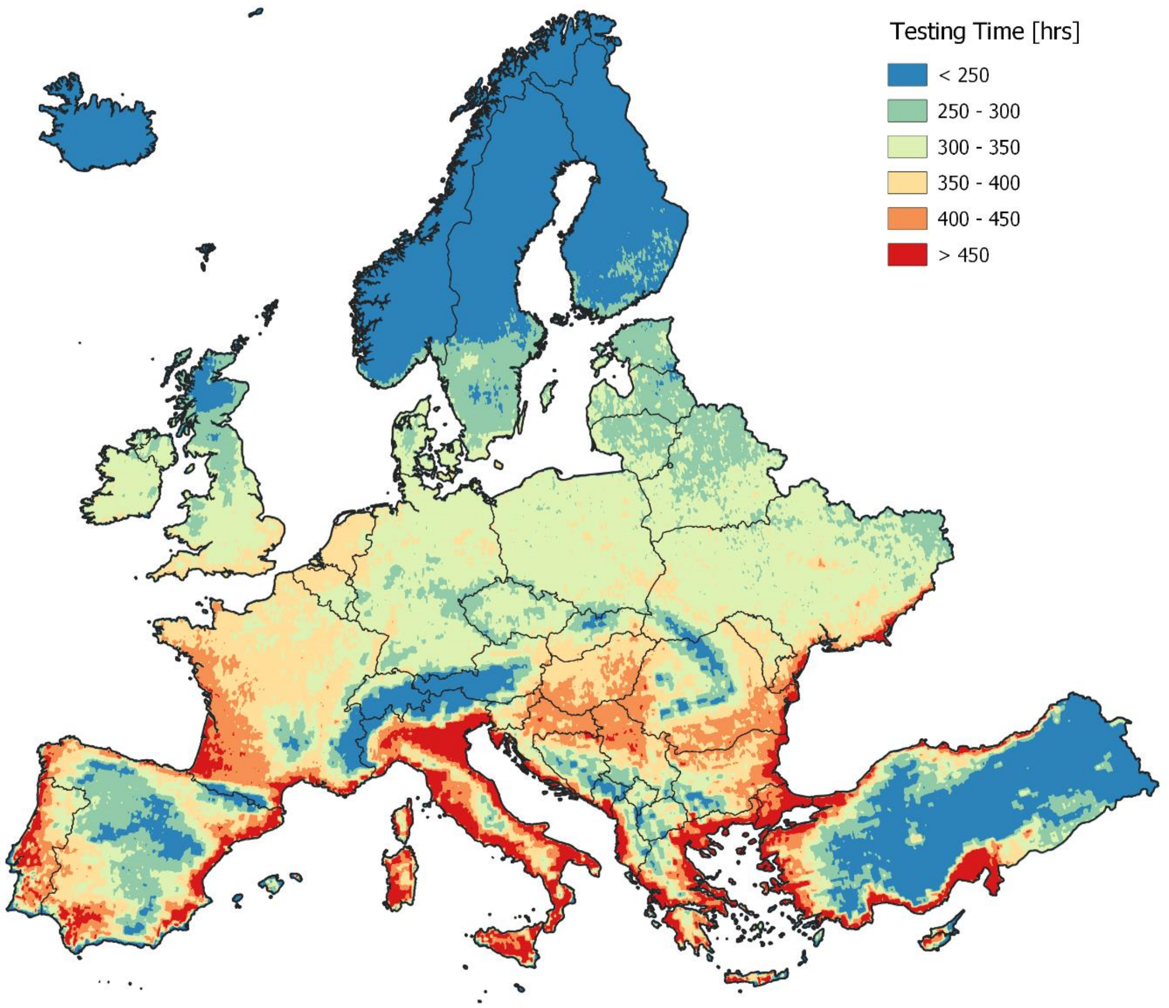

Figure S5. Equivalent damp heat testing time map of Europe for a service lifetime of 30 years for a module with an activation energy of 0.8 eV.

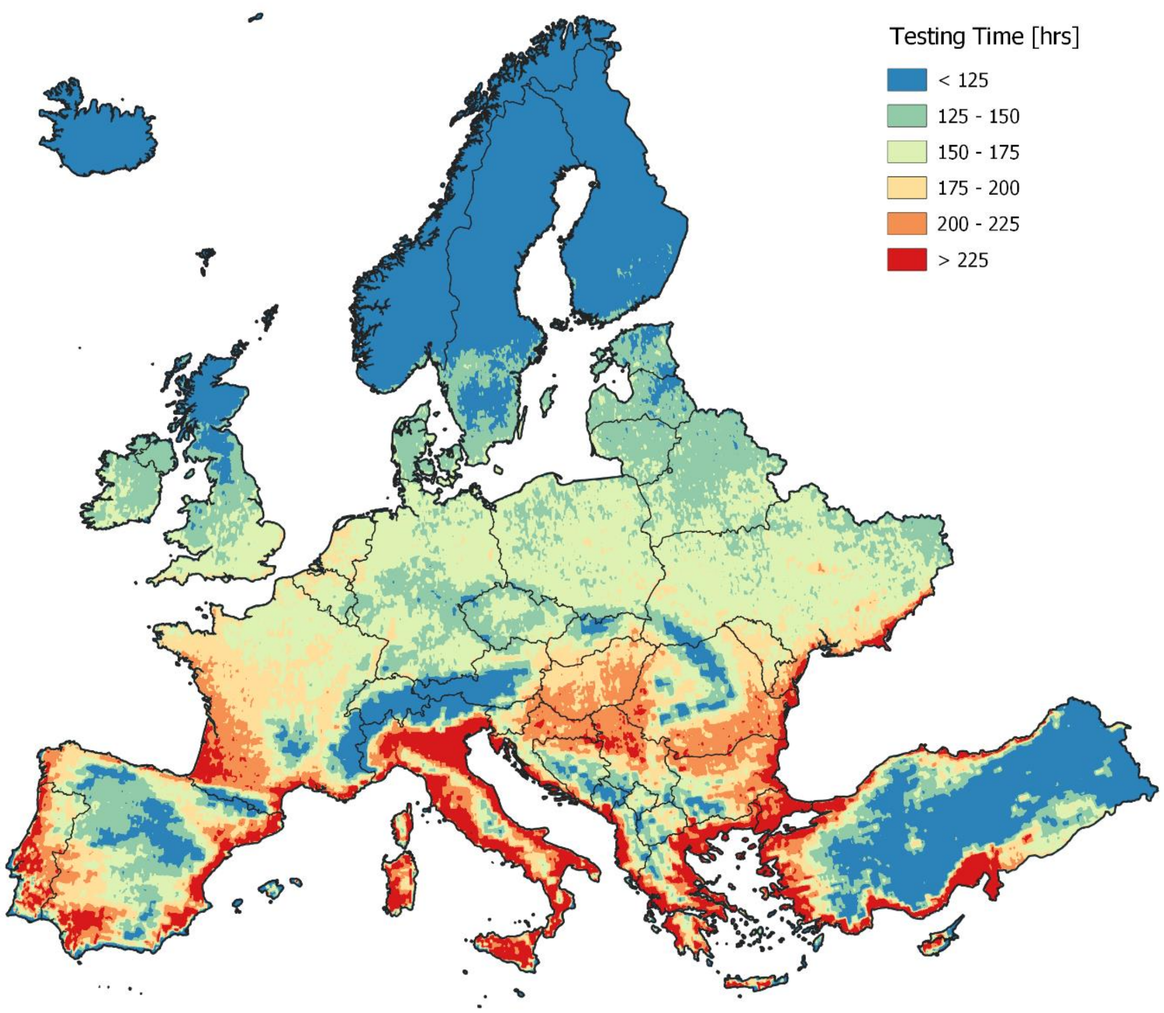

Figure S6. Equivalent damp heat testing time map of Europe for a service lifetime of 30 years for a module with an activation energy of 0.9 eV.

S4. Country-Specific Statistics on Damp Heat Testing Times

Table S3 reports country-specific statistics on damp heat testing times for a service lifetime of 30 years for a module with an activation energy of 0.6 eV. **Figure S7** visually illustrates the substantial variation in testing times, even within individual countries. These statistics were estimated based on pixel counts from the map in Figure 4 of the main manuscript. Additionally, **Figure S8** presents the spatial distribution of mean testing times across Europe.

Table S3. Country-specific statistics on damp heat testing times for a service lifetime of 30 years for a module with an activation energy of 0.6 eV.

Country	Mean [hrs]	Median [hrs]	Minimum [hrs]	Maximum [hrs]	Range [hrs]
Albania	1565	1502	911	2371	1460
Andorra	1042	1050	909	1148	239
Austria	1261	1327	529	1767	1238
Belarus	1399	1402	805	1610	805
Belgium	1639	1651	1314	1868	554
Bosnia and Herzegovina	1483	1456	1094	2145	1051
Bulgaria	1589	1620	855	2377	1521
Croatia	1694	1713	744	2448	1704
Czech Republic	1430	1429	1191	1640	449
Denmark	1460	1507	739	1790	1051
Estonia	1323	1332	626	1588	961
Finland	1028	1056	534	1492	958
France	1603	1639	562	2319	1757
Germany	1501	1511	717	1781	1064
Greece	1656	1670	386	2718	2333
Hungary	1652	1654	1327	1866	538
Iceland	768	764	434	1229	794
Ireland	1577	1604	620	1806	1186
Italy	1700	1765	344	2547	2202
Kosovo	1343	1353	945	1580	636
Latvia	1360	1361	756	1655	898
Liechtenstein	1067	1048	960	1194	234
Lithuania	1396	1395	845	1602	757

Luxembourg	1522	1519	1448	1595	148
Moldova	1513	1510	1365	1759	394
Monaco	998	672	175	1208	1033
Montenegro	1389	1249	978	2384	1407
North Macedonia	1324	1322	994	1816	822
Norway	914	937	515	1680	1165
Poland	1497	1500	781	1719	938
Portugal	1627	1654	391	2183	1792
Romania	1530	1556	1018	2285	1267
San Marino	1828	1775	1744	1967	223
Serbia	1597	1639	1112	1891	779
Slovakia	1457	1482	994	1755	761
Slovenia	1606	1625	1139	1989	850
Spain	1413	1377	426	2475	2049
Sweden	1024	961	466	1703	1238
Switzerland	1091	1069	484	1700	1215
The Netherlands	1726	1722	1402	1975	574
Turkey	1185	1054	322	3040	2719
Ukraine	1440	1433	640	2287	1647
United Kingdom	1497	1536	467	1942	1475

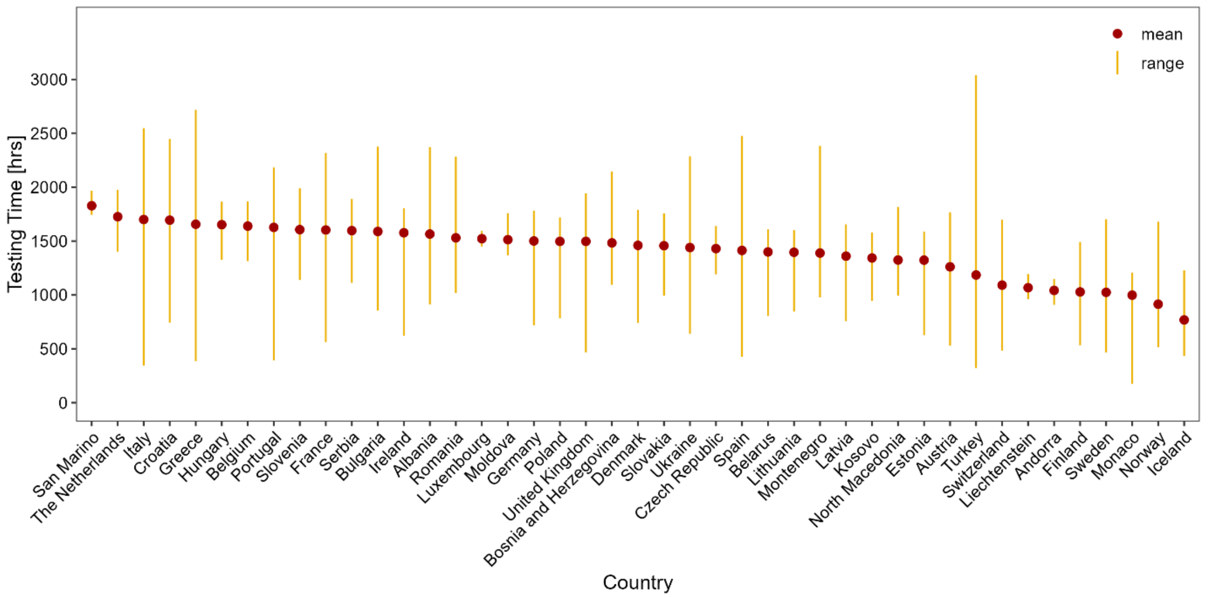

Figure S7. Country-specific statistics on damp heat testing times for a service lifetime of 30 years for a module with an activation energy of 0.6 eV, indicating the range of calculated testing times for each country with mean values arranged in descending order.

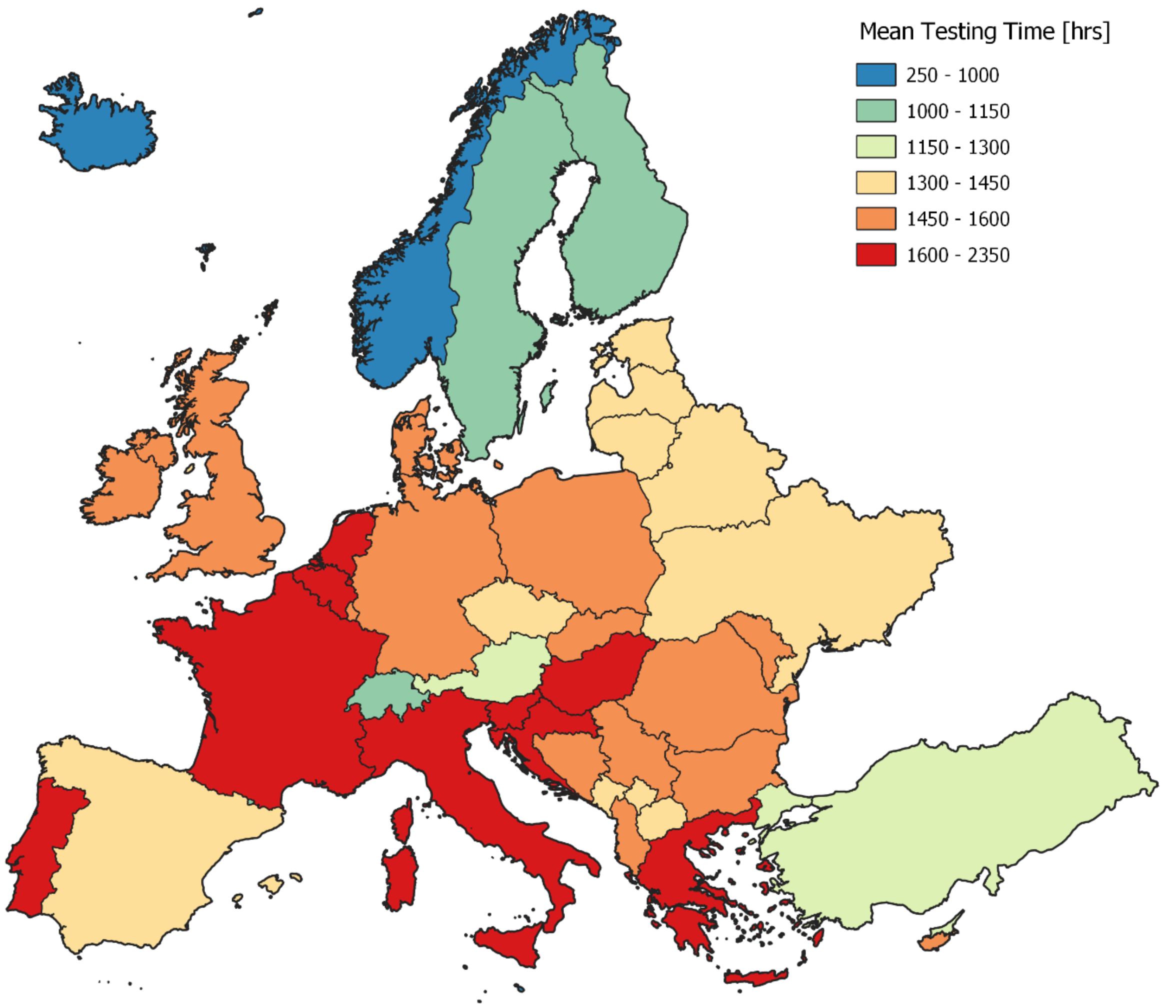

Figure S8. The spatial distribution of mean testing times across Europe for a service lifetime of 30 years for a module with an activation energy of 0.6 eV.

S5. Sensitivity Analysis Results on Parameter Estimations

Table S4 reports the sensitivity analysis results, emphasizing how the choice of exposure cut-off time during damp heat testing influences parameter estimation.

Table S4. Estimated model parameters depending on the damp heat exposure cut-off time.

Exposure Cut-off Time [hrs]	E_a [eV]	t_f [hrs]	m	n	A [hrs^{-1}]	R^2	MSE [hrs^2]	RMSE [hrs]
**2000**	0.790	127377.9	0.267	1.092	1335.4	0.959	1.32E-07	3.63E-04
**2500**	0.747	21310.0	0.437	1.326	1401.1	0.976	3.46E-07	5.88E-04
**3000**	0.719	8996.9	0.669	1.404	1400.0	0.984	1.01E-06	1.01E-03
**3500**	0.708	5786.2	0.980	1.464	1461.8	0.987	3.33E-06	1.83E-03
**4000**	0.699	4607.2	1.404	1.486	1449.2	0.988	1.17E-05	3.43E-03
**4500**	0.700	4140.9	1.970	1.525	1512.5	0.988	4.12E-05	6.42E-03
**5000**	0.695	3992.2	2.679	1.518	1471.8	0.987	1.30E-04	1.14E-02
**5500**	0.695	3988.4	3.452	1.521	1501.3	0.987	3.30E-04	1.82E-02
**6000**	0.697	4033.7	4.154	1.539	1536.2	0.987	6.28E-04	2.51E-02
**6500**	0.695	4080.9	4.688	1.524	1499.3	0.988	9.25E-04	3.04E-02
**7000**	0.694	4116.1	5.051	1.517	1493.2	0.989	1.14E-03	3.37E-02
**7500**	0.694	4139.3	5.279	1.518	1491.8	0.990	1.25E-03	3.54E-02
**8000**	0.694	4153.7	5.416	1.520	1491.0	0.991	1.29E-03	3.59E-02
**8500**	0.694	4162.5	5.498	1.521	1490.5	0.992	1.28E-03	3.57E-02
**9000**	0.694	4167.7	5.546	1.523	1490.3	0.993	1.24E-03	3.53E-02
**9500**	0.695	4170.9	5.575	1.524	1490.3	0.993	1.20E-03	3.46E-02
**10000**	0.700	4172.9	5.593	1.561	1495.2	0.994	1.15E-03	3.39E-02
